# Real-time reverse transcription PCR-based sequencing-independent pathotyping of Eurasian avian influenza A viruses of subtype H7

**DOI:** 10.1186/s12985-017-0808-3

**Published:** 2017-07-24

**Authors:** Annika Graaf, Martin Beer, Timm Harder

**Affiliations:** Friedrich Loeffler Institute, Institute of Diagnostic Virology, Südufer 10, Greifswald, 17493 Germany

**Keywords:** Avian influenza, Hemagglutinin subtype H7, Pathotyping, Real-time RT-PCR, Diagnosis, Cleavage site

## Abstract

Low pathogenic avian influenza viruses (LPAIV) of the subtypes H5 and H7 are known to give rise to highly pathogenic (HP) phenotypes by spontaneous insertional mutations which convert a monobasic trypsin-sensitive endoproteolytical cleavage site (CS) within the hemagglutinin (HA) protein into a polybasic subtilisin-sensitive one. Sporadic outbreaks of notifiable LPAIV H7 infections are continuously recorded in Europe and in Asia, and some lineages showed zoonotic transmission. De novo generation of HPAIV H7 from LPAIV precursors has been reported several times over the past decade. Rapid differentiation between LP and HP H7 virus strains is required as a prerequisite to emplace appropriate control measures. Here, reverse transcription real-time PCR assays (RT-qPCR) were developed and evaluated that allow LP and HP pathotype identification and distinction by probe-assisted detection of the HACS. These new RT-qPCRs allow a sensitive and highly specific pathotype identification of Eurasian subtype H7 AIV in allantoic fluids as well as in diagnostic field samples. RT-qPCR assisted pathotyping presents a rapid and sensitive alternative to pathotyping by animal inoculation or nucleotide sequencing.

**Electronic supplementary material:**

The online version of this article (doi:10.1186/s12985-017-0808-3) contains supplementary material, which is available to authorized users.

## Background

Avian influenza viruses (AIV) are members of the family *Orthomyxoviridae*, specified as influenza virus type-A*.* These viruses are further classified by the serologically defined subtypes of the predominant viral surface glycoproteins, the hemagglutinin (HA) and neuraminidase (NA) [1]. Their genome is composed of single-stranded, negative-sense RNA and comprises eight genome segments which encode at least ten proteins [2]. All 16 HA and nine NA AIV subtypes can be detected in populations of aquatic wild birds which form the natural reservoir of these viruses [3].

Based on their pathogenicity in chickens, two phenotypes of AIV are distinguished: highly pathogenic (HP) AIV and AIV of low pathogenicity (LPAIV). In nature, HP phenotypes have been restricted to viruses of subtypes H5 and H7. HPAIV arises from LPAI precursor viruses by spontaneous mutations leading to the insertion of basic amino acids into the cleavage site (CS) of the hemagglutinin protein (HA) which renders the HACS processible to subtilisin-like host proteases that are ubiquitous in all host tissues. Such viruses, therefore, gain competence for fatal systemic infections in avian hosts. LPAIV, in contrast, depends on local provision of trypsin-like proteases at the epithelial surfaces of the respiratory and/or gastrointestinal tracts and per se do not cause severe clinical signs [4]. All LPAIV and HPAIV infections of subtypes H5 and H7 in poultry are notifiable to the World Organization for Animal Health (O.I.E.). [5] Determination of the type of HACS is of utmost importance for the diagnosis of these infections. This can be achieved biologically by determination of the intravenous pathogenicity index (IVPI) in experimentally inoculated chickens or molecularly by nucleotide sequence analysis of the site encoding the HACS [6]. Since animal experiment facilities or expensive equipment are required for either pathway, solutions for alternative techniques have been sought in the past: These included restriction enzyme cleavage patterns [7], probe hybridization [8] and real time RT-PCR (RT-qPCR) approaches [9]. Based on the widespread availability of RT-qPCR technology in diagnostic laboratories and its recent favorable use in pathotyping of HPAIV H5 of the goose/Guangdong (gs/GD) lineage [10], this study was conducted to develop and validate sequencing-independent RT-qPCRs for pathotyping of Eurasian H7 AI viruses.

Over the past two decades, several incursions into poultry of subtype H7 LPAIV as well as the de novo generation and (in one case) spread of H7 HPAI viruses have been reported from Europe (Table 1). Other H7 LPAIV lineages have arisen in Eastern Asia, and one of them (H7N9/China) showed significant zoonotic propensities in annual waves of poultry-to-human transmission with more than 550 fatal human cases [11, 12]. Recently, the H7N9 lineages has also yielded an HP mutant which is spreading in southern China [13]. Considering the annual presence of LPAIV of subtype H7 in Eurasian wild bird populations [14] risks of new incursions into poultry in Europe are perpetuating.

**Table 1:** Outbreaks in poultry of subtype H7 avian influenza viruses of low (LP) and high (HP) pathogenicity in Europe, 1999–2016 [20]

Year	Country	Subtype	Pathotype	Number of infected holdings
1999–2000	Italy	H7N1	HP	1
2003	Netherland	H7N7	HP	255
2008	United Kingdom	H7N7	HP	1
2009	Germany	H7N7	LP	1
2009/2010	Spain	H7N7	LP/ HP	1/1^a^
2011	Germany	H7N7	LP	23
2013	Denmark	H7N7	LP	1
2013	Italy	H7N7	HP	6
2015	United Kingdom	H7N7	LP/ HP	1/1
2015	Germany	H7N7	LP/ HP	1/1
2015	Netherland	H7N7	LP	2
2016	Denmark	H7N7	LP	1
2016	Italy	H7N7	HP	2

^a^ Slash indicates that a matching pair of LP precursor and HP mutant viruses had been detected

## Methods

Based on the alignments of the HA H7 gene of a comprehensive selection of sequences from LP (*n* = 60) and HPAIV (*n* = 21) of Eurasian origin collected over the last decade in sequence databases (GenBank at NCBI; EpiFlu of the Global Initiative on Sharing Avian Influenza Data (GISAID)), a set of six primers was designed (Table 2). The selected primers targeted a short fragment of the HA gene that spans the endoproteolytic CS region [15–17]. The primers were designed for the broadest possible reactivity with recent Eurasian H7 sequences.

**Table 2:** Primers and probes used for sequencing-independent pathotyping of Eurasian avian influenza A subtype H7 viruses by real time RT-PCR

Primer/Probe ID	Sequence (5′ to 3′)	Location	Amplicon size	Accession number^a^
H7_CS-F1	TGMTGCTRGCAACAGGAAT	989–1007	107^b^	KX979524
H7_CS-F2 N	TGCTACTRGCAACAGGGAT	989–1007		
H7_CS-F3	TGMTGCTGGCAACWGGRAT	968–986		
H7_CS-R1N	CGTCAATKAGRCCTTCCCA	1096–1078		
H7_CS-R2N	TCCATTTTCWATRAAACCYGC	1056–1036		
H7_CS-R3	CATCAAYCAGACCYTCCCA	1056–1076		
H7_CS-LP-FAM	C + C + AAAG + GGA + A + GAG + GC	1026–1040		KY676327.1
H7_CS-HP_EMS-FAM	CCAAAGAGAAAGAGAAGAGGCC	1027–1046	120^c^	AB438941
H7_CS-HP_IT-FAM	TTCCAAAAGGATCGCGTGTGAGGA	1004–1027		KF493066

^a^Accession number of sequence/virus used to position the oligonucleotide along the HA gene

^b^size applied to LP sequences

^c^size applied to HP sequences

+ indicates that the following position constituted a “locked” nucleotide (LNA)

**Fig. 1:**
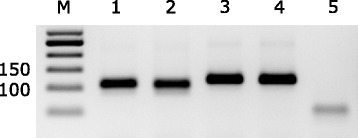
PCR-products generated to distinguish between low and high pathogenic avian influenza viruses of subtype H7. Primers listed in Table 2 were used for amplification. M - DNA size marker (50 bp ladder); 1 - LP H7N7 (A/mute swan/Germany/R901/2006); 2 - LP H7N9 (A/Anhui/1/2013); 3 – HP H7N1 (A/broiler/Italy/445/1999); 4 - HP H7N1 (A/chicken/Germany/AR1385/2015); 5 - LP H5N2 (A/teal-Foehr/Wv1378–79/2003) used as negative control

For validation of the assays, viral RNA from reference H7 LPAIV and HPAIV was used. Moreover, non-H7 influenza subtypes H5 and H9 as well as other avian respiratory viruses (infectious bronchitis virus (IBV), Newcastle disease virus (NDV)) were tested (Table 3). Viral RNA was purified with the QIAamp®Viral RNA Mini Kit (Qiagen, Hilden Germany) according to the manufacturer’s instructions. Primers were first evaluated in conventional RT-PCRs. The PCR reactions were carried out on a CFX96 thermocycler machine (Bio-Rad) using the following temperature profile: 30 min at 50 °C (RT), 2 min at 94 °C (inactivation of reverse transcriptase/activation of *Taq* polymerase), followed by 42 cycles of 30 s at 94 °C (denaturation), 30 s at 56 °C (annealing), and 30 s at 68 °C (elongation). Twenty-five μL per reaction were prepared using the SuperScript III One-Step RT-PCR system with Platinum *Taq* DNA polymerase (Invitrogen, Carlsbad, CA): For one reaction, 6.5 μl of RNase-free water, 12.5 μl reaction mix (2×), 1 μl of SuperScript III RT/Platinum *Taq*, and 5 μl of template RNA were mixed. Pre selected primers were then screened for their specifity using non H7-subtypes. Amplificates of the expected sizes were generated from both LP and HP phenotypes of subtype H7 viruses by conventional RT-PCR and visualized on an 2% agarose gel (Fig. 1).

**Table 3:** Analytical performance characteristics of real time RT-PCR (RT-qPCR) assays for sequencing-independent pathotyping of Eurasian reference H7 viruses

Reference virus	Accession number of HA	Sub- and pathotype	RT-qPCR method		
			LPAI H7	HPAI H7 ‘Emsland’	HPAI H7 ‘Italy’
A/mute swan/Germany/R901/2006	EPI359695	LP H7N7	Pos	Neg	Neg
A/Anhui/1/2013	AHZ60096	LP H7N9	Pos	Neg	Neg
A/chicken/Germany/AR1385/2015	SA	HP H7N7	Neg	Pos	Neg
A/broiler/Italy/445/1999	AJ580353	HP H7N1	Neg	Neg	Pos
A/turkey/Germany/R2025/2008	SA	LP H5N3	Neg	Neg	Neg
A/turkey/Germany/AR2485–86/2014	EPI552746	HP H5N8	Neg	Neg	Neg
A/chicken/Egypt/AR753–14/2013	EPI557457	HP H9N2	Neg	Neg	Neg
A/chicken/Sudan/AR251–15/2014	KX272465	IBV	Neg	Neg	Neg
A/chicken/Egypt/AR254–15/2014	SA	NDV	Neg	Neg	Neg

*LP* low pathogenic, *HP* high pathogenic, *Neg* negative, *Pos* positive, *SA* sequence available from the authors, also represented in the alignment in Additional file 1: Figure S1, *IBV* Infectious bronchitis virus, *NDV* Newcastle disease virus

Having assured the broad but exclusive specificity of the selected primers for Eurasian H7 viruses, matching probes for use in the RT-qPCR assays were developed. Initially, probes were designed with the aim to universally differentiate between LP and HP Eurasian H7 CS sequences. Probes were therefore placed directly across the sequence stretch encoding the CS. Closer inspection of the alignments, and taking into account also the list of HP H7 CS sequences provided by OFFLU [6], revealed that HP H7 CS sequences of Eurasian origin viruses were highly divergent: Viruses of separate outbreaks and epizootics represented unique CS sequences with little homology to viruses of other outbreaks. Within an outbreak series, however, HP H7 CS sequences proved to be conserved. This situation is opposed to HPAI H5 viruses of the gs/GD lineage which show considerable conservation even across different clades and allowed designing of a universal conserved probe for the HP phenotype of these viruses [10]. In contrast to HP H7, the HA CS of LP H7 viruses of Eurasian origin origin appeared to be fairly conserved [6]. Therefore, two strategies were followed to prove that sequencing-independent pathotyping by RT-qPCRs is principally possible also for Eurasian H7 viruses:

1. For HP H7, probes were designed that are specific for viruses of distinct outbreaks. Two distinct HP H7 outbreaks were selected: Isolates from a historic epizootic (Italy 1999, H7N1) and from the most recent HP H7 outbreak in Germany (referred to as ‘Emsland’; a region in the Northwest of Germany where a very high density of poultry population is reared) affecting a single holding in 2015 (H7N7) were chosen and specific Taqman probes matching the HA CS consensus sequences of each of these outbreaks designed (Table 2).
2. For Eurasian LP H7 a universal probe was developed and several universal Taqman probes were synthesized for comparison.

The same PCR conditions as described above for conventional RT-PCR were used for RT-qPCR, however, 2 μL of the RNAse-free water were replaced by 2 μl specific primer-probe mix. The HP mixes were composed of 1,25 pmol probe/μl and 3,75 pmol/μl for each forward and reverse primer.

## Results

**Fig. 2:**
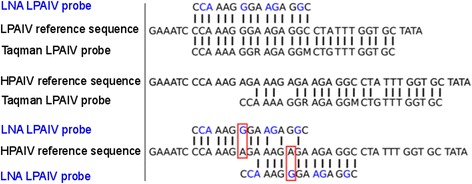
Alignment of probes within the hemagglutinin gene segment site encoding the cleavage site of low pathogenic (LP, upper panel) and highly pathogenic (HP, lower two panels) Eurasian avian influenza viruses of subtype H7. Upper panel shows perfect binding of an LNA probe (LNA positions in blue color) and of a conventional Taqman probe specific for LP H7 viruses. Central panel shows the same conventional Taqman probe binding to (and cross reacting with) a Eurasian HP H7 sequence. Lower panel shows hybridization of the LNA probe to an HP H7 ‘Emsland’ sequence; two hybridization positions are possible: The locked ‘G’ mismatch placed in the centre of the probe (red box) disabled binding and cross reaction at each of the two hybridization sites

Specificity was initially confirmed only for the two HP probes which specifically reacted with their homologous sequences but did not cross react with LP H7 or other HP H7 viruses (Table 3). The standard Taqman LP probes, however, did not sharply distinguish between pathotypes and cross reacted with various HP H7 viruses (not shown). Closer inspection of the alignments revealed that a single G/A mutation in the HA CS distinguished between LP and HP pathotypes (Fig. 2). Consequently, an LNA probe was designed placing the critical nucleotide position at the centre of the respective probe. Using this probe at a concentration of 2,5 pmol in the reaction mix finally allowed clear-cut distinction between LP and HP pathotypes by RT-qPCR (Table 3).

**Fig. 3:**
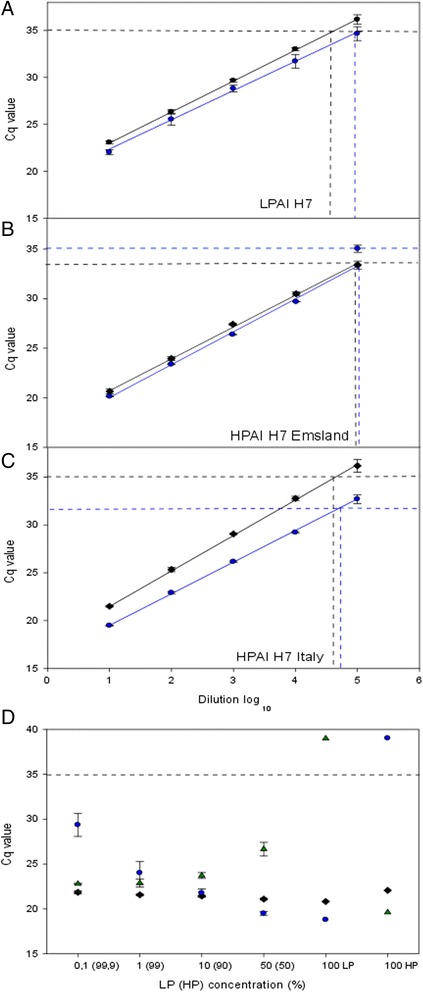
Determination of the limit of detection of three newly developed RT-qPCRs for sequencing-independent pathotyping of Eurasian avian influenza H7 viruses (blue dots/lines) compared to a matrix gene-specific generic RT-qPCR (Hoffmann et al., 2010; black diamonds/lines). The detection limit was determined based on serial ten-fold dilutions using RNA of the reference viruses (**a**) A/chicken/Germany/AR1385/2015 (HPAIV H7N7), (**b**) A/mute swan/Germany/R901/2006 (LPAIV H7N7) and (**c**) A/broiler/Italy/445/1999 (HPAIV H7N1). **d** Detection of artificial mixtures of H7 LP and HPAIV RNA of the ‘Emsland’ outbreak compared to a matrix gene-specific generic RT-qPCR (black diamonds). RNA of the reference viruses A/chicken/Germany/AR915/2015 (LPAIV H7N7) and A/chicken/Germany/AR1385/2015 (HPAIV H7N7) were mixed and the percentage ratios indicated on the X-axis. Identification of Cq values (results of triplicate analyses) obtained for each mixture sample by H7 specific RT-qPCRs is as follows: blue circles – LPAI H7; green triangles – HPAI H7 ‘Emsland’

The detection limit of the H7 pathotyping RT-qPCRs was determined by testing ten-fold serial dilutions of viral RNAs extracted from representative H7 LPAI and HPAI viruses. Average values of three independent runs were used for comparisons to a generic RT-qPCR for the M gene of these viruses [18]. A standard curve of each assay was generated showing a linear relationship between the log dilution of the viral RNA and the cycle quantification (Cq) value for both the specific and the generic assays (Fig. 3a-c). Considering the universal LP as well as the ‘Emsland’-specific HP probe, no significant difference between the median Cq values of each specific assay and the M RT-qPCR was found indicating that the newly developed and the generic RT-qPCRs have a similar analytical sensitivity. ﻿In contrast, the RT-qPCR detecting the historic Italian H7 HP lineage showed slightly higher sensitivity than the generic M RT-qPCR.

Furthermore, we determined the ability of the H7 pathotyping RTqPCRs to detect mixtures of RNAs of LPAIV and HPAIV derived from the Emsland outbreak in Germany, 2015, and compared it to the M gene-specific generic RT-qPCR (Fig. 3d). Different concentrations of LP/HPAIV-mixtures (0, 0.1%, 1%, 10%, 50% and 100% LP) were generated, and HP H7 RNA was added to 100%. Both RNA species were detected by the specific RT-qPCRs in the mixtures, and the respective Cq values reflected the concentration of the RNA species in the mixtures (Fig. 3d). H7 LP RNA was not detected in the sample containing 100% H7 HP RNA, and vice versa, once more confirming the specificity of the pathotyping RT-qPCRs (Fig. 3d). Thus, these PCRs can be used to study the generation and co-circulation of H7 HPAIV from its LPAI precursor viruses.

Assessment of the diagnostic performance characteristics of the established RT-qPCRs was carried out with a collection of H7 AIV isolates (*n* = 48) and H7-positive field samples (*n* = 27) collected between 1999 and 2016. Samples were obtained from the virus repository of the German National Reference Laboratory for Avian Influenza at the Friedrich-Loeffler-Institut, Germany, or kindly provided by the OIE Reference Laboratory for Newcastle Disease and Avian Influenza in Italy, ISZVe, Padua, the Central Veterinary Research laboratory at Dubai, United Arab Emirates, the National Centre for Foreign Animal Disease, Winnipeg, Canada and the WHO Collaborating Centre, London, United Kingdom, under the patronage of the global influenza programme (Table 4). Amplificates produced from these viral RNAs by H7-specific RT-qPCR analysis were also further processed for sequence analysis using the H7-specific reverse primer mix (Table 2) for Sanger sequencing: Following agarose gel electrophoresis and amplicon purification using the QIAquick Gel Extraction Kit (Qiagen, Hilden, Germany) they were cycle-sequenced (BigDye Terminator v1.1 Cycle Sequencing Kit, Applied Biosystems, California, United States) and analysed on an ABI PRISM 3130 Genetic Analyzer (Life Technologies, Darmstadt, Germany) [10]. Partial HA sequences of the diagnostic samples are shown in the sequence alignment of Additional file 1: Figure S1; in all cases subtype H7 was confirmed. Pathotypes were assigned as based on the deduced amino acid sequence of the HACS according to the list of published H7 CS sequences (Additional file 1: Figure S1 and Table 4).

**Table 4:** Diagnostic performance characteristics of the H7 pathotyping RT-qPCRs using HP and LP influenza A subtype H7 virus isolates and field samples collected from different countries and poultry holdings or wild bird species, 1999–2016

No.	Sample ID	Type of sample	Accession Number^a^	Subtype/ pathotype	PCR results (cq value)			
					M1.2	LP H7	HP H7 Italy	HP H7 Ems
1	A/duck/Potsdam/15/1980	I	AJ704797	H7N7 LP	17,03	NEG	NEG	NEG
2	A/duck/Potsdam/13/1980	I	SA	H7N7 LP	17,55	NEG	NEG	NEG
3	A/swan/Potsdam/64/1981	I	AM922155	H7N7 LP	20,07	NEG	NEG	NEG
4	A/turkey/Germany/R11/2001	I	AJ704812	H7N7 LP	18,89	12,74	NEG	NEG
5	A/mallard/NVP/1776–80/2003	I	NAV	H7N3 LP	25,3	16,41	NEG	NEG
6	A/mallard/NVP/41/2004	I	SA	H7N1 LP	15,44	12,49	NEG	NEG
7	A/mallard/Föhr/Wv190/2005	I	NAV	H7N7 LP	27,35	24,10	NEG	NEG
8	A/teal/Föhr/Wv180/2005	I	NAV	H7N2 LP	14,28	10,76	NEG	NEG
9	A/teal/Föhr/Wv177/2005	I	AM933237	H7N7 LP	24,41	21,76	NEG	NEG
10	A/mallard/Germany/R721/2006	I	SA	H7N7 LP	31,38	27,31	NEG	NEG
11	A/graylag goose/Germany/R752/2006	I	AM933236	H7N7 LP	26,15	17,27	NEG	NEG
12	A/mallard/Germany/R756/2006	I	SA	H7N4 LP	24,81	24,13	NEG	NEG
13	A/mute swan/Germany/R57/2006	I	EPI492518	H7N7 LP	27,73	24,20	NEG	NEG
14	A/mute swan/Germany/R901/2006	I	EPI359695	H7N1 LP	23,14	20,08	NEG	NEG
15	A/swan/Germany/736/2006	I	EPI492517	H7N4 LP	15,39	14,07	NEG	NEG
16	A/common pochard/Germany/R916/2006	I	SA	H7N7 LP	19,03	20,32	NEG	NEG
17	A/duck/Germany/R3129/2007	I	SA	H7N7 LP	15,34	11,59	NEG	NEG
18	A/sentinel-duck/Germany/SK207R/2007	I	NAV	H7N3 LP	27,64	22,09	NEG	NEG
19	A/mallard/Sko212-219 K/2007	I	SA	H7N3 LP	25,97	21,04	NEG	NEG
20	A/guineafowl/Germany/R2495/2007	I	AM930528	H7N3 LP	29,58	27,14	NEG	NEG
21	A/mallard/Germany/R192/2009	I	SA	H7N7 LP	14,65	13,28	NEG	NEG
22	A/turkey/Germany/R655/2009	F	EPI302173	H7N7 LP	13,34	11,76	NEG	NEG
23	A/nandu/Germany/AR142/2013	F	SA	H7N7 LP	28,35	28,90	NEG	NEG
24	A/turkey/Germany/AR502/2013	F	SA	H7N7 LP	18,67	19,12	NEG	NEG
25	A/turkey/Germany/AR618/2013	F	NAV	H7Nx LP	16,11	16,20	NEG	NEG
26	A/chicken/Germany/AR909/2013	F	SA	H7Nx LP	35,59	NEG	NEG	NEG
27	A/turkey/Germany/AR979/2013	F	NAV	H7Nx LP	25,59	21,79	NEG	NEG
28	A/environment/Germany/AR1251/2013	F	NAV	H7N LP	21,31	14,93	NEG	NEG
29	A/chicken/Germany/AR929/2015	F, EL	SA	H7N7 LP	30,39	30,02	NEG	NEG
30	A/chicken/Germany/AR930/2015	F, EL	SA	H7N7 LP	30,39	35,77	NEG	NEG
31	A/chicken/Germany/AR934/2015	F, EL	SA	H7N7 LP	30,07	32,88	NEG	NEG
32	A/chicken/Germany/AR943/2015	F, EL	SA	H7N7 LP	30,07	32,70	NEG	NEG
33	A/chicken/Germany/AR944/2015	F, EL	SA	H7N7 LP	30,07	31,03	NEG	NEG
34	A/chicken/Germany/AR945/2015	F, EL	SA	H7N7 LP	29,9	33,18	NEG	NEG
35	A/chicken/Germany/AR946/2015	F, EL	SA	H7N7 LP	29,9	33,32	NEG	NEG
36	A/duck/Germany/AR234/1/2016	F	SA	H7N7 LP	33,42	35,43	NEG	NEG
37	A/duck/Germany/AR2112/2016	F	NAV	H7N7 LP	36,17	*NEG*	NEG	NEG
38	A/duck/Germany/AR2868/2016	F	NAV	H7N7 LP	35,3	*NEG*	NEG	NEG
39	A/FPV/Rostock/45/1934	I	CY077420	H7N1 HP	17,25	NEG	NEG	13,94
40	A/chicken/Germany/“Taucha“/1979	I	SA	H7N7 HP	14,25	NEG	NEG	10,63
41	A/chicken/Germany/R28/2003	I	AJ704813	H7N7 HP	14,77	NEG	NEG	NEG
42	A/FPV/dutch/1927	I	NAV	H7N1 HP	16,52	NEG	NEG	32,14
43	A/chicken/Germany/AR1385/2015	F, EL	SA	H7N7 HP	18,76	NEG	NEG	19,01
44	A/chicken/Germany/AR1413/2015	F, EL	SA	H7N7 HP	29,9	NEG	*NEG*	35,48
45	A/chicken/Germany/AR1488/1/2015	F, EL	SA	H7N7 HP	29,31	NEG	NEG	22,72
46	A/environment/Germany/AR1536/2015	F, EL	SA	H7N7 HP	29,38	NEG	NEG	21,18
47	A/environment/Germany/AR1537/2015	F, EL	SA	H7N7 HP	29,38	NEG	NEG	25,7
48	A/environment/Germany/AR1539/2015	F, EL	SA	H7N7 HP	29,38	NEG	NEG	22,19
49	A/environment/Germany/AR1540/2015	F, EL	SA	H7N7 HP	29,38	NEG	NEG	24,69
50	A/environment/Germany/AR1541/2015	F, EL	SA	H7N7 HP	29,38	NEG	*NEG*	26,35
51	A/environment/Germany/AR1546/2015	F, EL	SA	H7N7 HP	30,12	NEG	NEG	25,19
52	A/turkey/Italy/472/1999	I	AJ704811	H7N1 LP	15,24	9,80	NEG	NEG
53	A/chicken/Italy/473/1999	I	EPI624438	H7N2 LP	13,73	10,71	NEG	NEG
54	A/turkey/Italy/2043/2003	I	CY022613, CY022615	H7N3 LP	24,34	21,45	NEG	NEG
55	A/duck/Italy/636/2003	I	NAV	H7N3 LP	22,05	20,49	NEG	NEG
56	A/chicken/Brescia/19/2002	I	AM922154	H7N1 HP	16,59	NEG	*NEG*	NEG
57	A/hen/Italy/444/1999	I	AJ704810	H7N1 HP	16,22	NEG	18,02	NEG
58	A/broiler/Italy/445/1999	I	AJ580353	H7N1 HP	17,02	NEG	16,35	NEG
59	A/turkey/Ireland/PV8/1995	I	AJ704799	H7N7 LP	16,19	13,07	NEG	NEG
60	A/houbara/Dubai/AR433/2014	I	SA	H7N1 LP	16,81	13,51	NEG	NEG
61	A/houbara/Dubai/AR434/2014	I	SA	H7N1 LP	14,67	11,27	NEG	NEG
62	A/houbara/Dubai/AR435/2014	I	SA	H7N1 LP	15,47	12,66	NEG	NEG
63	A/houbara/Dubai/AR436/2014	I	SA	H7N1 LP	12,23	9,23	NEG	NEG
64	A/houbara/Dubai/AR437/2014	I	SA	H7N1 LP	16,1	13,35	NEG	NEG
65	A/houbara/Dubai/AR438/2014	I	SA	H7N1 LP	13,71	10,08	NEG	NEG
66	A/peregrine falcon/Dubai/AR439/2014	I	SA	H7N1 LP	13,79	26,82	NEG	NEG
67	A/francolin/Dubai/AR440/2014	I	SA	H7N2 LP	15,85	17,84	NEG	NEG
68	A/wild bird/Dubai/AR3452/2014	F	SA	H7N1 LP	16,18	14,57	NEG	NEG
69	A/alexandria tyrode/T145/1948	I	SA	H7N1 HP	14,48	NEG	NEG	10
70	A/duck/Alberta/48/1976	I	SA	H7N3 LP	15,8	14,08	NEG	NEG
71	A/turkey/Ontario/18–1/2000	I	AF497552	H7N1 LP	28,61	NEG	NEG	NEG
72	A/mallard/Alberta/8734/2007	I	AM933238	H7N3 LP	18,63	NEG	NEG	NEG
73	A/chicken/BritishColumbia/CN-06/2004	I	KP055066	H7N3 HP	16,42	NEG	NEG	NEG
74	A/chicken/BritishColumbia/CN-07/2004	I	KP055076	H7N3 HP	24,71	NEG	NEG	NEG
75	A/Anhui/1/2013	I	AHZ60096	H7N9 LP	11,79	9,94	NEG	NEG

^a^Sequences were obtained from the EpiFlu database of the Global Initiative on Sharing Avian Influenza Data (GISAID) and from GenBank at the National Center for Biotechnology Information (NCBI)

*LP * low pathogenicity, *HP* high pathogenicity, *SA* sequence shown in either Additional file 1: Figure S1 or Additional file 2: Figure S2, otherwise accession numbers are indicated, *NAV* sequence not available, *neg* no positive signal detected, *I* Isolate, *F* Field sample, *F, EL* field sample from recent outbreak in Germany

**Fig. 4:**
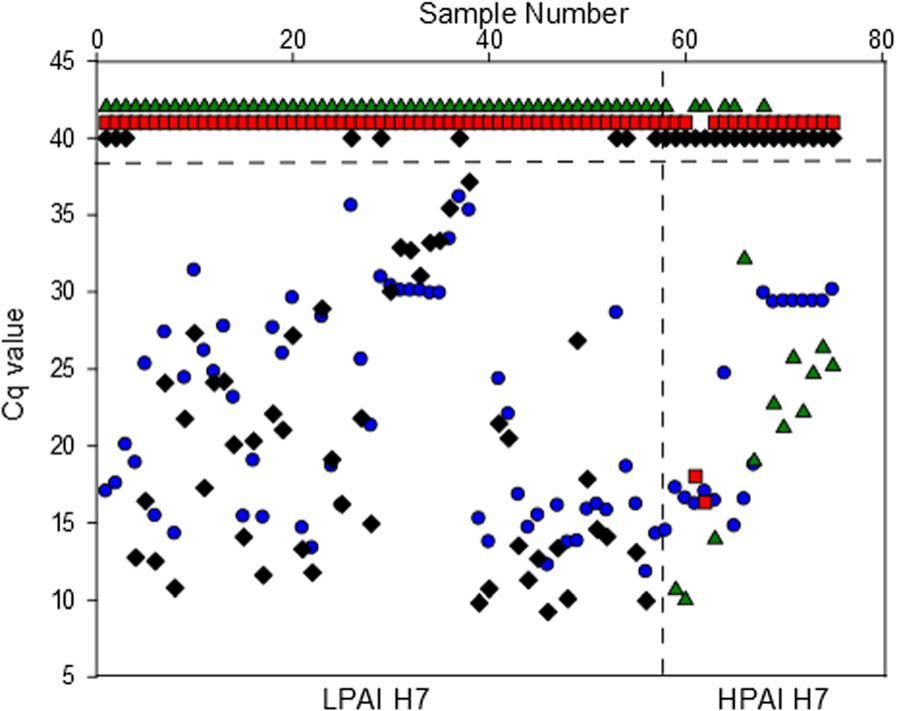
Sequencing-indpendent pathotyping of isolates and clinical samples of avian influenza subtype H7 viruses by real-time RT-PCRs (RT-qPCR). Sample numbers refer to the identification of viruses in Table 4. Cq values generated for each sample by the influenza A virus-generic M1.2 RT-qPCR are depicted as blue dots. Identification of Cq values obtained for each sample by H7 specific RT-qPCRs is as follows: black diamonds – LPAI H7; green triangles – HPAI H7. ‘Emsland’; red squares – HPAI H7 ‘Italy’

In total, 75 samples positive for AIV of subtype H7 were used. Based on nucleotide sequence analysis and/or IVPI, 49 samples were classified as LPAIV and 15 as HPAIV (Table 4, Additional file 1: Figure S1). They were of both historic and recent origin and mainly derived from European locations. Four samples originated from North America, nine from the United Arab Emirates/Dubai and one represented the Chinese LP H7N9 lineage. The samples mainly consisted of egg-derived isolates or native combined oropharyngeal and cloacal swabs obtained from poultry or wild birds. Seven samples were taken from the environment during a recent HPAIV outbreak in a chicken layer holding in Germany (referred to as ‘Emsland’). For the H7 LP RT-qPCR, 48 out of 56 samples were correctly identified as LP (Table 4, Fig. 4), also including the Chinese LP H7N9 reference virus. Three historic LP isolates (Table 4, nos. 1–3) and the two North American LP H7 viruses (Table 4, nos. 71–72) were not detected despite high viral loads. Sequence mismatches affected binding of either probe and/or primers in these cases. In three further samples (Table 4, nos. 26, 37, 38) low virus loads were detected by the generic M RT-qPCR and these were missed by the H7 LP specific RT-qPCR. However, in most samples, the H7 LP specific RT-qPCR proved to be more sensitive as compared to the generic M specific one (Table 4, Fig. 4). Since none of the HP H7 positive samples cross reacted in the H7 LP RT-qPCR, complete specificity was achieved.

A total of 19 samples harbored HP H7 RNA. None of them was detected by the LP specific RT-qPCR (Table 4, Fig. 4). Two isolates originating from the Italian HP H7N1 epizootic of 1999 were detected by the H7 HP ‘Italy’-specific RT-qPCR (Table 4, nos. 57–58); no further viruses were identified by this PCR. This includes another HPAIV H7N1 isolate from Italy originating from 2002 and distinguished from the 1999 viruses by 13 mutations in the primer and probe binding sites (Table 4, no. 56). Thus, the ‘Italy 99’ RT-qPCR proved to be highly lineage-specific. The second H7 HP RT-qPCR aimed at detecting HP AIV related to the most recent outbreak in Germany in 2015. All nine samples classified to harbor HP H7 were identified by this PCR with a high sensitivity (Table 4, nos. 43–51). At similarily high sensitivity four historic European HP H7 viruses (Table 4, nos. 39, 40, 42, 69), but none of the Italian HP viruses, or an isolate (Table 4, no. 41) representing the large HP H7N7 epizootic affecting the Netherlands, Belgium and Germany in 2003, reacted with either of the two HP specific RT-qPCRs. No cross reactivity to any of the LP H7 samples was detected indicating excellent performance values regarding sensitivity and specificity. Due to our results, the threshold distinguishing reliably between positive and negative samples was set at Cq = 38.

## Discussion

Although not all of the LP and HP H7 samples did show a positive signal with the respective RT-qPCR due to mismatches in the probe binding regions, the newly developed set of primers produced a sequenceable amplificate even of those virus strains. Consequently, pathotype confirmation of a H7 positive sample that tested negative by the LP and HP RT-qPCRs is still possible by nucleotide sequence analysis using the amplificate produced by these RT-qPCRs. In this respect, the newly developed RT-qPCRs resemble the one introduced by Slomka et al. [19] which also spanned the H7 HACS but its probe targeted a highly conserved sequence stretch outside the CS.

## Conclusion

The pathotype-specific RT-qPCRs developed here for avian influenza viruses of subtype H7 proved to be a useful, sensitive and highly specific alternative to nucleotide sequence analysis for the characterization of LPAI and HPAI H7 viruses of European origin. Proper detection of HP H7 viruses required knowledge of the HACS of the specific lineage, and specific probes are to be used for each distinct lineage. Thus, initial characterization of an H7 HP virus still depends on nucleotide sequence analysis of its HACS. However, in case of on-going spread of the identified HP H7 lineage a lineage-specific probe can then be used in a pathotyping RT-qPCR for the swift examination and pathotyping of further cases and outbreaks. Furthermore, the LP LNA probe introduced here was universally usuable for Eurasian LP H7 viruses circulating in Europe over the past decade. In conclusion, these here described RT-qPCRs complement a sequencing-independent approach, and allow a high-speed pathotyping helping the authorities to install necessary control measures in time.

## Additional file

Additional file 1: Nucleotide sequences encoding the HA endoproteolytic cleavage site of H7N7 low pathogenic avian influenza viruses generated within this study. (PDF 90 kb)
Additional file 2: Nucleotide sequences encoding the HA endoproteolytic cleavage site of H7N7 highly pathogenic avian influenza viruses generated within this study. (PDF 57 kb)

## Abbreviations

AIV: Avian influenza virus
CS: Cleavage site
GISAID: Global initiative on sharing Avian influenza data
gs/GD: Goose/Guangdong
HA: Hemagglutinin
HPAIV: Highly pathogenic avian influenza virus
IBV: Infectious bronchitis virus
IVPI: Intravenous pathogenicity index
LPAIV: Low pathogenic avian influenza virus
NA: Neuraminidase
NDV: Newcastle disease virus
RT-qPCR: Reverse transcription real-time PCR
