# Supplementary material for: Real-time reverse transcription PCR-based sequencing-independent pathotyping of Eurasian avian influenza A viruses of subtype H7
Source: Virol J. 2017 Jul 24;14:137. doi: 10.1186/s12985-017-0808-3 (PMC5525275; doi:10.1186/s12985-017-0808-3)
Supplement: Additional file 1: — Nucleotide sequences encoding the HA endoproteolytic cleavage site of H7N7 low pathogenic avian influenza viruses generated within this study. (PDF 90 kb) [file 12985_2017_808_MOESM1_ESM.pdf]

```
                                          1        10        20        30        40        50        60
                                          |        |         |         |         |         |         |
            A/duck/Potsdam/13/1980        TATGTCCGAGATATGTTAAGCAAGAGAGCCTGCTACTGGCAACAGGGATGAAGAACGTTC
            A/swan/Potsdam/64/1981        TATGTCCGAGATATGTTAAGCAAGAGAGCCTGCTACTGGCAACAGGGATGAAGAACGTTC
               A/avian/R224/2010          TATGTCCGAGATATGTTAAGCAAGAGAGCCTGCTACTGGCAACAGGGATGAAGAACGTTC
  A/commonpochard/Germany/R916/2006       TATGTCCGAGATATGTTAAGCAGGAGAGTCTGATGCTGGCAACAGGAATGAAGAATGTTC
        A/mallard/Alberta/8734/2007       TATGTCCGAGATATGTTAAGCAAGAGAGCCTGCTACTGGCAACAGGGATGAAGAACGTTC
        A/chicken/Germany/AR909/2013      -ATGTCCGAGATATGTTAAGCAGGAGAGTCTGATGCTAGCAACCGGAATGAAAAATGTTC
         A/chicken/Germany/AR915/2015     -----------TATGTTAAGCAGGAGAGTCTGATGCTAGCAACCGGAATGAAAAACGTTC
         A/chicken/Germany/AR925/2015     TATGTCCGAGATATGTTAAGCAGGAGAGTCTGATGCTAGCAACCGGAATGAAAAACGTTC
        A/chicken/Germany/AR934/2015      TATGTCCGAGATATGTTAAGCAGGAGAGTCTGATGCTAGCAACCGGAATGAAAAACGTTC
        A/chicken/Germany/AR942/2015      -----------TATGTTAAGCAGGAGAGTCTGATGCTAGCAACCGGAATGAAAAACGTTC
        A/chicken/Germany/AR943/2015      TATGTCCGAGATATGTTAAGCAGGAGAGTCTGATGCTAGCAACCGGAATGAAAAACGTTC
       A/chicken/Germany/AR944/2015       TATGTCCGAGATATGTTAAGCAGGAGAGTCTGATGCTAGCAACCGGAATGAAAAACGTTC
         A/Mallard/Germany/R192/2009      TATGTCCGAGATATGTTAAGCAGGAAAGTCTGATGCTGGCAACAGGAATGAAAAATGTTC
           A/duck/Germany/R3129/2007      TATGTCCGAGATATGTTAAGCAGGAGAGTCTGATGCTGGCAACAGGAATGAAAAATGTTC
  A/Greylag goose/Germany/R752/2006       TATGTCCGAGATATGTTAAGCAGGAGAGTTTGATGCTGGCAACAGGAATGAAGAATGTTC
         A/mallard/Germany/R756/2006      TATGTCCGAGATATGTTAAGCAGGAGAGTTTGATGCTGGCAACAGGAATGAAGAATGTTC
              A/Teal/Föhr/Wv177/2005      TATGTCCGAGATATGTTAAGCAGGAGAGTCTGATGCTGGCAACAGGAATGAAGAATGTTC
        A/turkey/Germany/R655-5/2009      TATGTCCGAGATATGTTAAGCAGGAGAGTCTGATGCTGGCAACAGGAATGAAGAATGTTC
         A/francolin/Dubai/AR440/2014     TATGTCCGAGATATGTTAAGCAGGAGAGTCTGATGCTGGCAACAGGAATGAAGAATGTTC
         A/houbara/Dubai/AR438/2014       TATGTCCGAGATATGTTAAGCAGGAGAGTCTGATGCTGGCAACAGGAATGAAGAATGTTC
A/Peregrine falcon/Dubai/AR439/2014       TATGTCCGAGATATGTTAAGCAGGAGAGTCTGATGCTGGCAACAGGAATGAAGAATGTTC
        A/duck/Germany/AR234/1/2016       -----------TATGTTAAGCAGGAGAGTCTGATGCTGGCAACAGGAATGAAAAATGTTC
          A/nandu/Germany/AR142/2013      -----------TATGTTAAGCAGGAGAGTCTGATGCTGGCAACAGGAATGAAAAATGTTC
              A/duck/Alberta/48/1976      TATGTCCGAGATATGTTAAGCAGGAGAGTCTGATGCTGGCAACAGGAATGAAGAATGTTC
          A/houbara/Dubai/AR433/2014      AATGTCCGAGATATGTTAAGCAGGAGAGTCTGATGCTGGCAACAGGAATGAAGAATGTTC
          A/houbara/Dubai/AR434/2014      AATGTCCGAGATATGTTAAGCAGGAGAGTCTGATGCTGGCAACAGGAATGAAGAATGTTC
          A/houbara/Dubai/AR435/2014      AATGTCCGAGATATGTTAAGCAGGAGAGTCTGATGCTGGCAACAGGAATGAAGAATGTTC
         A/houbara/Dubai/AR436/2014       AATGTCCGAGATATGTTAAGCAGGAGAGTCTGATGCTGGCAACAGGAATGAAGAATGTTC
         A/houbara/Dubai/AR437/2014       TATGTCCGAGATATGTTAAGCAGGAGAGTCTGATGCTGGCAACAGGAATGAAGAATGTTC
          A/mallard/Sko212-219K/2007      TATGTCCGAGATATGTTAAGCAGGAGAGTCTGATGCTGGCAACAGGAATGAAGAATGTTC
              A/mallard/NVP/41/2004       TATGTCCGAGATATGTTAAGCAAGAGAGTCTGCTGCTGGCAACAGGAATGAAGAATGTTC
         A/mallard/Germany/R721/2006      ------------------------------------------------------------
                                          61       70        80        90        100       110       120
                                          |        |         |         |         |         |         |
            A/duck/Potsdam/13/1980        CTGAAATTCCAAAAGGGAGAGGACTATTTGGTGCCATAGCGGGTTTTATTGAAAATGGGT
            A/swan/Potsdam/64/1981        CTGAAATTCCAAAAGGGAGAGGACTATTTGGTGCCATAGCGGGTTTTATTGAAAATGGGT
               A/avian/R224/2010          CTGAAATTCCAAAAGGGAGAGGACTATTTGGTGCCATAGCGGGTTTTATT-AAAATGGGT
  A/commonpochard/Germany/R916/2006       CCGAAATCCCAAAGGGAAGAGGCCTATTTGGTGCTATAGCGGGTTTCATTGAAAATGGAT
        A/mallard/Alberta/8734/2007       CTGAAATTCCAAAAGGGAGAGGACTATTTGGTGCCATAGCGGGTTTTATTGAAAATGGGT
        A/chicken/Germany/AR909/2013      CTGAAATCCCAAAGGGAAGAGGCCTATTTGGTGCTATAGCGGGTTTCATTGAAAATGGAT
         A/chicken/Germany/AR915/2015     CTGAAATCCCAAAGGGAAGAGGCCTATTTGGTGCTATAGCGGGATTCATTGAAAATGGAT
         A/chicken/Germany/AR925/2015     CTGAAATCCCAAAGGGAAGAGGCCTATTTGGTGCTATAGCGGGATTCATTGAAAATGGAT
        A/chicken/Germany/AR934/2015      CTGAAATCCCAAAGGGAAGAGGCCTATTTGGTGCTATAGCGGGATTCATTGAAAATGGAT
        A/chicken/Germany/AR942/2015      CTGAAATCCCAAAGGGAAGAGGCCTATTTGGTGCTATAGCGGGATTCATTGAAAATGG--
        A/chicken/Germany/AR943/2015      CTGAAATCCCAAAGGGAAGAGGCCTATTTGGTGCTATAGCGGGATTCATTGAAAATGGAT
       A/chicken/Germany/AR944/2015       CTGAAATCCCAAAGGGAAGAGGCCTATTTGGTGCTATAGCGGGATTCATTGAAAATGGAT
         A/Mallard/Germany/R192/2009      CCGAACTCCCAAAGGGAAGAGGCCTATTTGGTGCTATAGCGGGTTTCATTGAAAATGGAT
           A/duck/Germany/R3129/2007      CTGAAATCCCAAAGGGAAGAGGCCTATTTGGTGCTATAGCGGGTTTCATTGAAAATGGAT
  A/Greylag goose/Germany/R752/2006       CCGAAATCCCAAAGGGAAGAGGCCTATTTGGTGCTATAGCGGGTTTCATTGAAAATGGAT
         A/mallard/Germany/R756/2006      CCGAAATCCCAAAGGGAAGAGGCCTATTTGGTGCTATAGCGGGTTTCATTGAAAATGGAT
              A/Teal/Föhr/Wv177/2005      CCGAAATCCCAAAGGGAAGAGGCCTATTTGGTGCTATAGCGGGTTTCATTGAAAATGGAT
        A/turkey/Germany/R655-5/2009      CCGAAATCCCAAAGGGAAGAGGCCTATTTGGTGCTATAGCGGGTTTCATTGAAAATGGAT
         A/francolin/Dubai/AR440/2014     CCGAACTCCCAAAGGGAAGAGGCCTATTTGGTGCTATAGCGGGTTTCATTGAAAATGGAT
         A/houbara/Dubai/AR438/2014       CCGAACTCCCAAAGGGAAGAGGCCTATTTGGTGCTATAGCGGGTTTCATTGAAAATGGAT
A/Peregrine falcon/Dubai/AR439/2014       CCGAACTCCCAAAGGGAAGAGGCCTATTTGGTGCTATAGCGGGTTTCATTGAAAATGGAT
        A/duck/Germany/AR234/1/2016       CCGAAATCCCAAAAGGAAGAGGCCTGTTTGGTGCTATAGCAGGTTTCATTGAAAATGGAT
          A/nandu/Germany/AR142/2013      CCGAAATCCCAAAAGGAAGAGGCCTGTTTGGTGCTATAGCAGGTTTCATTGAAAATG---
              A/duck/Alberta/48/1976      CCGAAATCCCAAAGGGAAGAGGCCTTTTTGGTGCTATAGCGGGTTTCATTGAAAATGATT
          A/houbara/Dubai/AR433/2014      CCGAACTCCCAAAGGGAAGAGGCCTATTTGGTGCTATAGCGGGTTTCATTGAAAATGGAT
          A/houbara/Dubai/AR434/2014      CCGAACTCCCAAAGGGAAGAGGCCTATTTGGTGCTATAGCGGGTTTCATTGAAAATGGAT
          A/houbara/Dubai/AR435/2014      CCGAACTCCCAAAGGGAAGAGGCCTATTTGGTGCTATAGCGGGTTTCATTGAAAATGGAT
         A/houbara/Dubai/AR436/2014       CCGAACTCCCAAAGGGAAGAGGCCTATTTGGTGCTATAGCGGGTTTCATTGAAAATGGAT
         A/houbara/Dubai/AR437/2014       CCGAACTCCCAAAGGGAAGAGGCCTATTTGGTGCTATAGCGGGTTTCATTGAAAATGGAT
          A/mallard/Sko212-219K/2007      CCGAACTCCCAAAGGGAAGAGGCCTATTTGGTGCTATAGCGGGTTTCATKAAAA--GGAT
              A/mallard/NVP/41/2004       CCGAAGTCCCAAAGGGAAGAGGCCTATTTGGTGCTATAGCGGGTTTCAGTGAGGATGGAT
         A/mallard/Germany/R721/2006      -CGAAATCCCAAAGGGAAGAGGCCTATTTGGTGCTATAGCAGGTTTCATTGAAAATGGAT
                                          121      130       140       150
                                          |        |         |         |      LPAI H7 probe
            A/duck/Potsdam/13/1980        GGGAAGGTCTGGTTGATGGATGGTAGGCTCA
            A/swan/Potsdam/64/1981        GGGAAGGTCTGGTTGATGGATGGTAGGCTCA
               A/avian/R224/2010          GGAAGGTACT-GTTGAT-GATGGTAGGCTCA
  A/commonpochard/Germany/R916/2006       GGGAAGGTCTTATTGACGGGTGGTAGGCTCA
        A/mallard/Alberta/8734/2007       GGGATATACTAGTTGATGGATGGTAGGCTCA
        A/chicken/Germany/AR909/2013      GGGAAGGTCTGATTGACGGATGGTAGGCTTC
         A/chicken/Germany/AR915/2015     -------------------------------
         A/chicken/Germany/AR925/2015     GGGAAGGCCTGATTGACGGATGGTAGGCTTC
        A/chicken/Germany/AR934/2015      GGGAAGGCCTGATTGACGGATGGTAGGCTCA
        A/chicken/Germany/AR942/2015      -----------------------------TA
        A/chicken/Germany/AR943/2015      GGGAAGGCCTGATTGACGGATGGTAGGCTCA
       A/chicken/Germany/AR944/2015       GGGAAGGCCTGATTGACGGATGGTAGGCTCA
         A/Mallard/Germany/R192/2009      GGGAAGGTCTGATTGACGGGTGGTAGGCTCA
           A/duck/Germany/R3129/2007      GGGAAGGTCTGATTGACGGGTGGTAGGCTCA
  A/Greylag goose/Germany/R752/2006       GGGAAGGTCTGATTGACGGGTGGTAGGCTCA
         A/mallard/Germany/R756/2006      GGGAAGGTCTGATTGACGGGTGGTAGGCTCA
              A/Teal/Föhr/Wv177/2005      GGGAAGGTCTGATTGACGGGTGGTAGGCTCA
        A/turkey/Germany/R655-5/2009      GGGAGGGTCTGATTGATGGGTGGTAGGCTCA
         A/francolin/Dubai/AR440/2014     GGGAAGGTCTGATTGACGGGTGGTAGGCTCA
         A/houbara/Dubai/AR438/2014       GGGAAGGTCTGATTGACGGGTGGTAGGCTCA
A/Peregrine falcon/Dubai/AR439/2014       GGGAAGGTCTGATTGACGGGTGGTAGGCTCA
        A/duck/Germany/AR234/1/2016       -------------------------------
          A/nandu/Germany/AR142/2013      -------------------------------
              A/duck/Alberta/48/1976      GGGGTTATCTGATTGACGGGTGGTAGGCTCA
          A/houbara/Dubai/AR433/2014      GGGAAGGTCTGATTGACGGGTGGTATGGCTT
          A/houbara/Dubai/AR434/2014      GGGAAGGTCTGATTGACGGGTGGTATGGCTT
          A/houbara/Dubai/AR435/2014      GGGAAGGTCTGATTGACGGGTGGTATGGCTT
         A/houbara/Dubai/AR436/2014       GGGAAGGTCTGATTGACGGGTGGTATGGCTT
         A/houbara/Dubai/AR437/2014       GGGAAGGTC-GATTGACGGGTGGTAGGCTCA
          A/mallard/Sko212-219K/2007      GGGAAGGTCTGATTGACGGGTGGTAGGCTCA
              A/mallard/NVP/41/2004       GGGAAGGTCTTATTGATGGTGGTAGGGCTCA
         A/mallard/Germany/R721/2006      GGAAGGCCCCTCTTGACGGGTGGTAGGCTCA
```
