# Supplementary material for: Real-time reverse transcription PCR-based sequencing-independent pathotyping of Eurasian avian influenza A viruses of subtype H7
Source: Virol J. 2017 Jul 24;14:137. doi: 10.1186/s12985-017-0808-3 (PMC5525275; doi:10.1186/s12985-017-0808-3)
Supplement: Additional file 2: — Nucleotide sequences encoding the HA endoproteolytic cleavage site of H7N7 highly pathogenic avian influenza viruses generated within this study. (PDF 57 kb) [file 12985_2017_808_MOESM2_ESM.pdf]

```
                                        1        10        20        30        40        50        60
                                        |        |         |         |         |         |         |
   A/chicken/Germany/"Taucha"/1979      AATGCCCCAGATATGTGAAACAAGAGAGCCTGCTACTGGCAACAGGGATGAAGAACGTTC
      A/Chicken/Germany/AR1385/2015     AATGTCCGAGATATGTTAAGCAGGAGAGTCTGATGCTAGCAACCGGAATGAAAAACGTTC
    A/chicken/Germany/AR1413/2015       TATGTCCGAGATATGTTAAGCAGGAGAGTCTGATGCTAGCAACCGGAATGAAAAACGTTC
  A/chicken/Germany/AR1488/1/2015       TATGTCCGAGATATGTTAAGCAGGAGAGTCTGATGCTAGCAACCGGAATGAAAAACGTTC
 A/environmen/Germany/AR1536/2015       TATGTCCGAGATATGTTAAGCAGGAGAGTCTGATGCTAGCAACCGGAATGAAAAACGTTC
 A/environment/Germany/AR1537/2015      TATGTCCGAGATATGTTAAGCAGGAGAGTCTGATGCTAGCAACCGGAATGAAAAACGTTC
A/environment/Germany/AR1539/2015       TATGTCCGAGATATGTTAAGCAGGAGAGTCTGATGCTAGCAACCGGAATGAAAAACGTTC
A/environment/Germany/AR1540/2015       TATGTCCGAGATATGTTAAGCAGGAGAGTCTGATGCTAGCAACCGGAATGAAAAACGTTC
A/environment/Germany/AR1541/2015       TATGTCCGAGATATGTTAAGCAGGAGAGTCTGATGCTAGCAACCGGAATGAAAAACGTTC
A/environment/Germany/AR1546/2015       TATGTCCGAGATATGTTAAGCAGGAGAGTCTGATGCTAGCAACCGGAATGAAAAACGTTC
                                        61       70        80        90        100       110       120
                                        |        |         |         |         |         |         |
   A/chicken/Germany/"Taucha"/1979      CTGAAATTCCAAAAAAGAAAAAGAAAAAGAGAGGCTTATTTGGTGCCATAGCGGGTTTTA
      A/Chicken/Germany/AR1385/2015     CTGAAATCCCA------AAGAGAAAGAGAAGAGGCCTATTTGGTGCTATAGCGGGATTCA
    A/chicken/Germany/AR1413/2015       CTGAAATCCCA------AAGAGAAAGAGAAGAGGCCTATTTGGTGCTATAGCGGGATTCA
  A/chicken/Germany/AR1488/1/2015       CTGAAATCCCA------AAGAGAAAGAGAAGAGGCCTATTTGGTGCTATAGCGGGATTCA
 A/environmen/Germany/AR1536/2015       CTGAAATCCCA------AAGAGAAAGAGAAGAGGCCTATTTGGTGCTATAGCGGGATTCA
 A/environment/Germany/AR1537/2015      CTGAAATCCCA------AAGAGAAAGAGAAGAGGCCTATTTGGTGCTATAGCGGGATTCA
A/environment/Germany/AR1539/2015       CTGAAATCCCA------AAGAGAAAGAGAAGAGGCCTATTTGGTGCTATAGCGGGATTCA
A/environment/Germany/AR1540/2015       CTGAAATCCCA------AAGAGAAAGAGAAGAGGCCTATTTGGTGCTATAGCGGGATTCA
A/environment/Germany/AR1541/2015       CTGAAATCCCA------AAGAGAAAGAGAAGAGGCCTATTTGGTGCTATAGCGGGATTCA
A/environment/Germany/AR1546/2015       CTGAAATCCCA------AAGAGAAAGAGAAGAGGCCTATTTGGTGCTATAGCGGGATTCA
                                        121      130       140       150       160
                                        |        |         |         |         |
   A/chicken/Germany/"Taucha"/1979      TTGAAAATGGGTGGGAAGGTCTGATTGA---------------
      A/Chicken/Germany/AR1385/2015     TTGAAAATGGATGGGAAGGCCTGATTGACGGATGGTATGGCTT
    A/chicken/Germany/AR1413/2015       TTGAAAATGGATGGGAAGGCCTGATTGACGGATGGTAGGCTCA
  A/chicken/Germany/AR1488/1/2015       TTGAAAATGGATGGGAAGGCCTGATTGACGGATGGTAGGCTCA
 A/environmen/Germany/AR1536/2015       TTGAAAATGGATGGGAAGGCCTGATTGACGGATGGTAGGCTCA
 A/environment/Germany/AR1537/2015      TTGAAAATGGATGGGAAGGCCTGATTGACGGATGGTAGGCTCA
A/environment/Germany/AR1539/2015       TTGAAAATGGATGGGAAGGCCTGATTGACGGATGGTAGGCTCA
A/environment/Germany/AR1540/2015       TTGAAAATGGATGGGAAGGCCTGATTGACGGATGGTAGGCTCA
A/environment/Germany/AR1541/2015       TTGAAAATGGATGGGAAGGCCTGATTGACGGATGGTAGGCTCA
A/environment/Germany/AR1546/2015       TTGAAAATGGATGGGAAGGCCTGATTGACGGATGGTAGGCTCA
```

HPAI H7 Emsland probe
